# The Hidden Cavernous Mystery Behind a Headache: A Case Report of Tolosa-Hunt Syndrome

**DOI:** 10.7759/cureus.95669

**Published:** 2025-10-29

**Authors:** Sreekanth Bommala, Mohammed Hady Albitar, Fatima K Zubair, Asad A Alnahar, Sahana Bopparaju

**Affiliations:** 1 Emergency Medicine, People’s Education Society Institute of Medical Sciences and Research, Kuppam, IND; 2 Internal Medicine, College of Medicine, Alfaisal University, Riyadh, SAU; 3 Ophthalmology, Osmania General Hospital, Hyderabad, IND; 4 General Medicine, Atlas International Medical Complex, Riyadh, SAU; 5 Internal Medicine, Osmania General Hospital, Hyderabad, IND

**Keywords:** case report, headache, idiopathic inflammation, ophthalmoplegia, ptosis, steroids, tolosa-hunt syndrome

## Abstract

Tolosa-Hunt syndrome (THS) is a rare, non-specific inflammatory disorder that typically manifests as painful ophthalmoplegia, associated with systemic symptoms such as gastrointestinal distress, and restricted eye movements. This case report describes a patient with acute neurological symptoms, including right-sided headache, eye pain, and oculomotor, trochlear, and abducens nerve palsy.

A 45-year-old female patient presented with a complaint of acute right-sided headache and eye pain. The patient exhibited significant oculomotor, trochlear, and abducens nerve palsies. All other physical and neurological examinations, as well as routine investigations, were normal. A brain MRI revealed noncaseating granulomatous inflammation consistent with the diagnosis of THS, which was responsible for the painful ophthalmoplegia.

The patient was treated with glucocorticoid therapy, which resulted in a drastic improvement of symptoms, including the resolution of the headache, eye pain, and nerve palsy.

THS is a rare diagnosis, often requiring exclusion of other potential causes of ophthalmoplegia. This case highlights the importance of considering THS in the differential diagnosis of patients presenting with acute cranial nerve palsies and unexplained headache, especially when imaging reveals granulomatous inflammation. Prompt treatment with glucocorticoids can lead to significant symptom resolution.

THS, though uncommon, should be considered in the differential diagnosis of patients with acute, unexplained ophthalmoplegia and headache. Early diagnosis and initiation of glucocorticoid therapy can significantly improve patient outcomes.

## Introduction

Headaches are among the most common neurological complaints that lead patients to seek medical help. International Classification of Headache Disorders (ICHD-3) gives the framework for the classification of causes of headaches [1]. They are mainly classified into primary headaches, which are unrelated to any underlying medical condition, and secondary headaches, which are due to vascular, infectious, neoplastic, and other medical conditions that increase the intracranial pressure [1].

An uncommon presentation of unilateral headache associated with ptosis and painful, restricted eye movements is Tolosa-Hunt syndrome (THS). The etiology is believed to be an idiopathic granulomatous inflammatory process, with the potential to spread into the superior orbital fissure and cavernous sinus [2]. The cranial nerve palsies of the III, IV, and VI cranial nerves are also associated with THS. The pathogenesis of this condition involves an autoimmune mechanism and has been reported in association with systemic lupus erythematosus and sarcoidosis [3,4].

The early and definitive diagnosis of THS is critical as the standard treatment protocol involving the use of high-dose steroids provides symptomatic relief and prevents long-term morbidity. The use of corticosteroids is followed by a gradual tapering of the dosage over several months or longer, depending on the specific case [2]. This case report has been prepared in accordance with the 2023 CARE (CAse REport) guidelines [5].

## Case presentation

Case history/examination

A 45-year-old female was admitted to the hospital with a history of unilateral headache and pain in the right eye for 20 days. She also gave a history of drooping of her right eyelids and double vision for 18 days.

She was asymptomatic until 20 days ago, when she developed the above symptoms while attending an event. The patient reported an acute onset of unilateral headache, localized to the right side of the head, which was severe in intensity and non-pulsatile. The headache followed an intermittent pattern, occurring in about two episodes per day over the past 20 days, and was more frequent during the night. Each episode of headache lasted for about 30 minutes and was associated with nausea and vomiting. The headache resolved following each episode of vomiting. It was also associated with phonophobia. There was no history of photophobia, tearing, nasal stuffiness, facial flushing, or change in severity on lying down. The unilateral right-sided headache was associated with right-sided eye pain, which lasted for the same duration as the episode of headache. The eye pain was described as a boring type with blurring of vision.

She managed the episodes herself using paracetamol and nonsteroidal anti-inflammatory drugs (NSAIDs), along with home remedies such as ice packs, which provided only symptomatic relief. She did not seek any medical consultation during the 20 days prior to hospitalization. Despite taking these measures, the symptoms worsened progressively.

The patient’s daughter observed drooping of the patient’s right eyelid, which was followed two days later by restricted movement of the right eye. The patient also reported experiencing double vision in the same eye. There was no history of trauma or similar complaints involving the left eye. The eyelid drooping did not improve with rest, and there was no history of eye protrusion.

The patient gave a history of decreased perception of sensation over the right side of her face while washing her face 20 days back. The patient complained of feeling a hot and cold sensation, albeit to a lesser extent, on the right side of the face. The patient had a prior significant history of deviation of the mouth to the right side for 12 years, which was secondary to Bell’s palsy. The patient could perceive smell and taste normally and had no history of difficulty in chewing and swallowing. There was no history of decreased salivation, lacrimation, dysarthria, increased sensitivity to noise, vertigo, or tinnitus. The patient was able to move her tongue sufficiently to clear impacted food particles and also had no associated difficulty in turning her head to either side. The patient was assessed for neurological function through relevant history, and it was found to be unremarkable. There was no autonomic involvement or cortical motor involvement. Meningeal signs and cerebellar signs were negative. There was no history of recent vaccinations, dog bites, ear discharge, or OCP use. The patient did not exhibit any other comorbidities.

**Figure 1:**
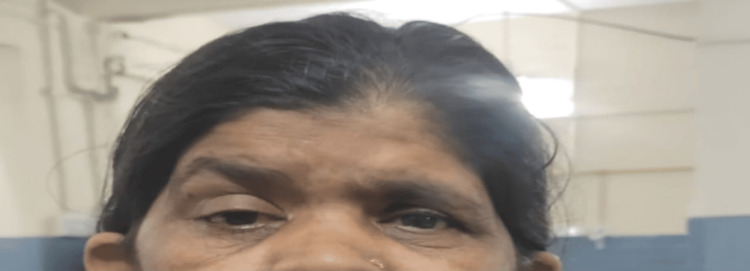
Clinical signs of right optic nerve involvement and ophthalmoplegia Consent was obtained to publish the image.

The patient was alert, cooperative, coherent, and well-oriented to time, place, and person. On evaluation, her vital signs were within the normal range. Neurological examination revealed normal higher mental functions, with a Mini-Mental Status Examination score of 28/30. Right eye visual acuity was 5/60, which improved to 6/24 with the use of a pinhole. There was moderate ptosis of the right upper eyelid, accompanied by brow lift, and restriction of extraocular muscle movements in all directions. No deviation of the eyeball was observed, and the pupil was approximately 3 mm, sluggishly reactive to light. Accommodation was absent in the right eye, and no nystagmus was detected, indicating possible involvement of the right oculomotor nerve (Figure 1).

Examination of the left eye showed visual acuity of 6/6. Assessment of the cranial nerves revealed paresis of the right oculomotor, trochlear, and abducens nerves, along with sensory deficits in the ophthalmic and maxillary divisions of the right trigeminal nerve. Examination of the facial nerve showed reduced facial movements on the left side, with impaired ability to wrinkle the forehead on the left. Taste sensations were normal. Direct and indirect light reflexes of both eyes were tested and found to be sluggish in the right eye, consistent with the involvement of the right oculomotor nerve. No optic nerve damage was observed, and all other neurological examinations were normal. Physical and systemic examination revealed no other abnormalities.

Differential diagnosis

To differentiate between these conditions, the following diagnostic tests were conducted: MRI of the brain, including contrast-enhanced MRI/CT angiography, to rule out space-occupying lesions, tumors, vascular malformations, or inflammatory processes; cerebrospinal fluid (CSF) analysis to check for signs of infection or inflammation; optical coherence tomography (OCT) to assess for optic nerve damage; blood tests including complete blood count (CBC), inflammatory markers, and autoimmune panels to identify underlying systemic conditions; and electromyography (EMG) and nerve conduction studies (NCS) to evaluate cranial nerve function and rule out inflammatory neuropathies or demyelinating diseases.

Investigations

On investigation, the hematology report showed hemoglobin of 11 g/dL, red blood cells of 4.2 million/µL, white blood cell count of 6500/µL, and platelet count of 335,000/µL. Biochemical laboratory investigations revealed low sodium at 130 mEq/L, elevated alkaline phosphatase of 105 U/L, and an elevated erythrocyte sedimentation rate of 32 mm. Blood glucose, HbA1c, serum urea, serum creatinine, serum electrolytes, and all other microbiological investigations were within normal limits.

CSF analysis showed a clear appearance, normal glucose levels, normal protein levels, and a cell count of 3 cells, all of which were lymphocytes. CSF adenosine deaminase levels were 4 U/L, which fall under the normal range and are not suggestive of tuberculosis (TB). CSF culture, Gram stain, and GeneXpert results were negative. Investigations for diabetes mellitus-related hypoglycemia and stroke were also ruled out by relevant investigations and history.

**Figure 2:**
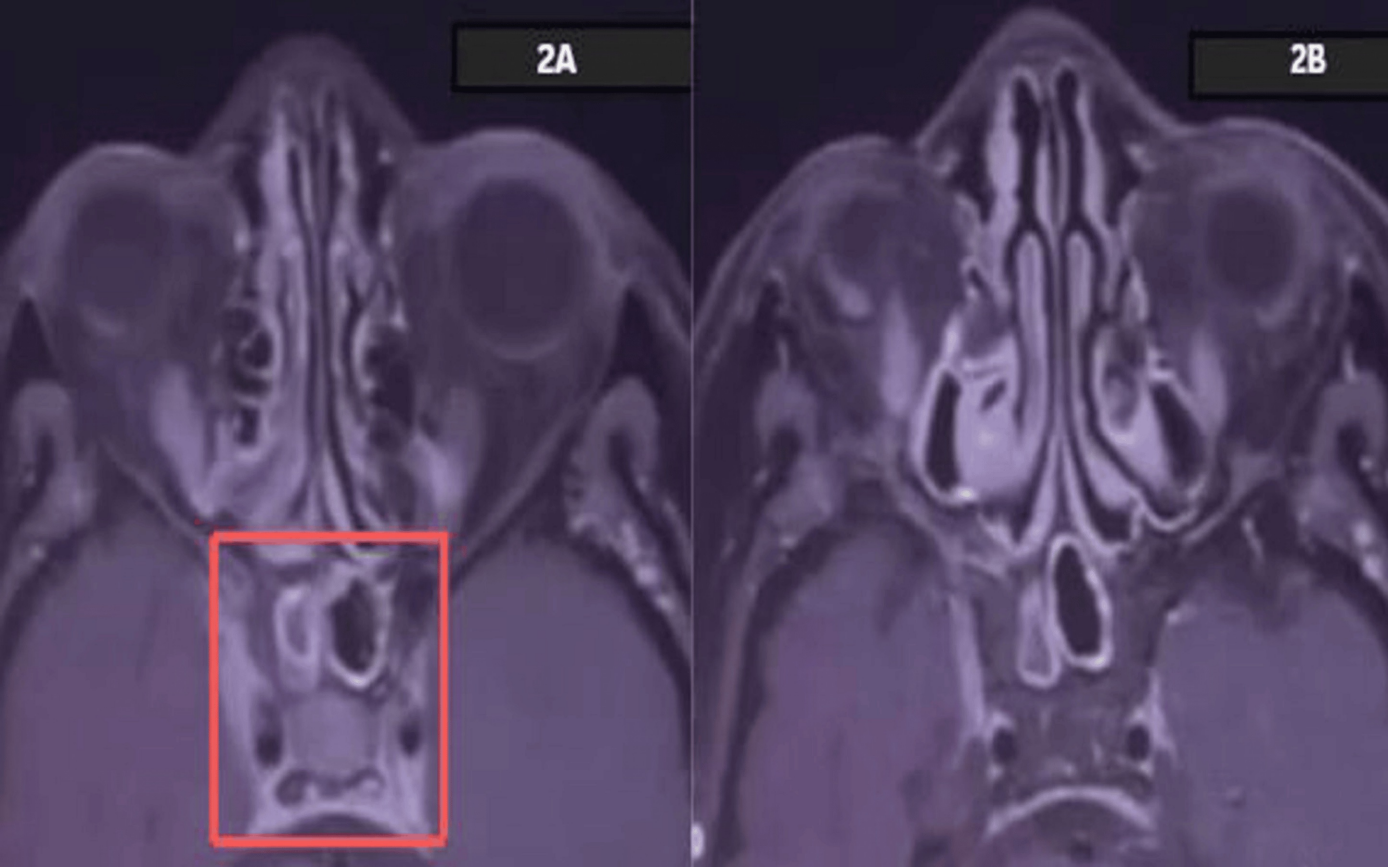
Axial MRI of the brain showing a hyperintense soft tissue lesion in the right cavernous sinus region 2A: MRI scan showing the coronal view of the nasal and paranasal structures. The image highlights the sinus cavities. 2B: MRI scan showing the coronal view of the nasal and paranasal structures with a slightly different contrast or setting, emphasizing the structural features in the sinus area.

**Figure 3:**
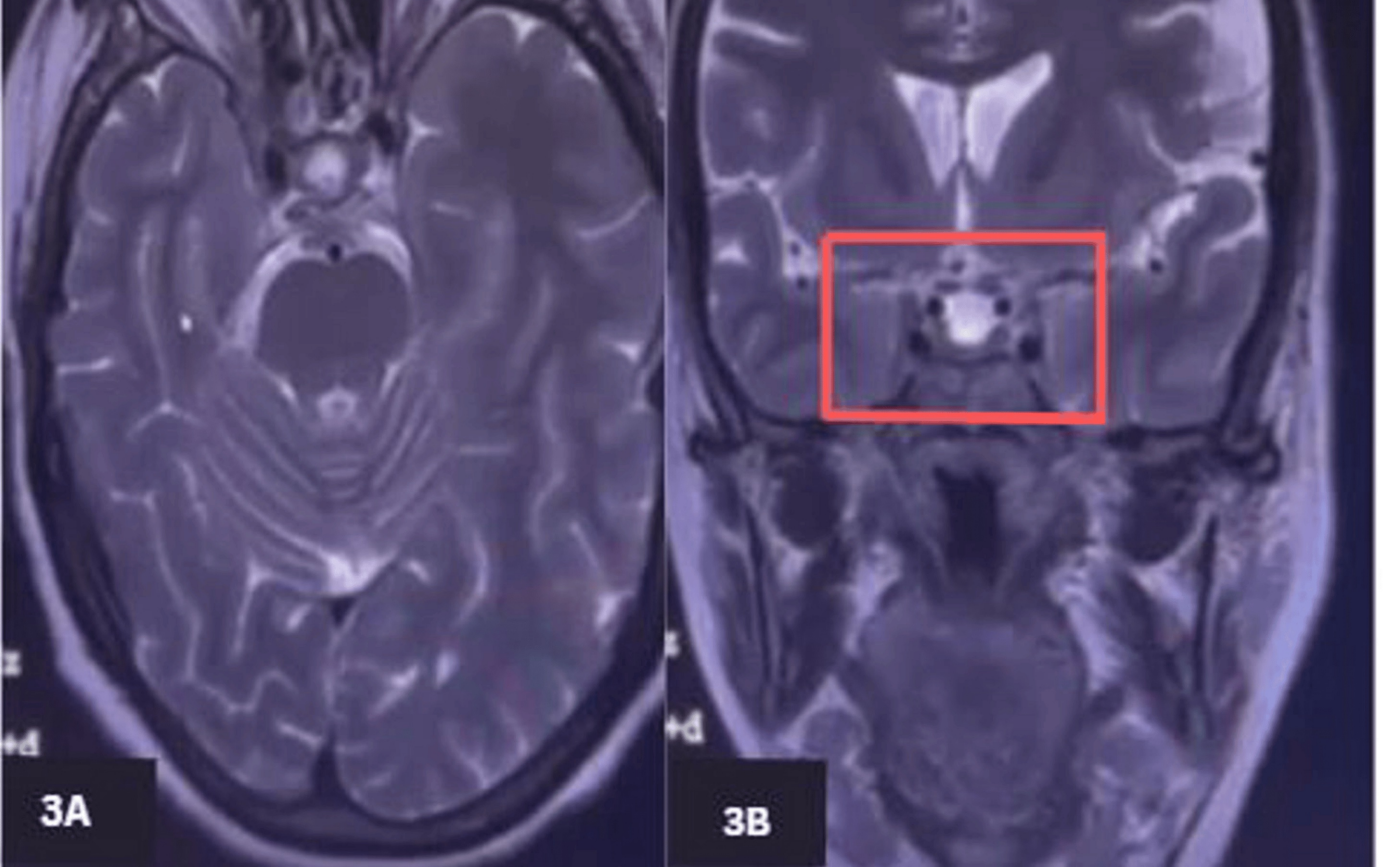
Post-contrast MRI of the brain demonstrating homogeneous enhancement of a lesion involving the right cavernous sinus, orbital fissures, and optic canal 3A: Axial MRI scan of the head showing midline brain structures, including the optic nerves and surrounding regions, typically used to assess ocular and cerebral abnormalities. 3B: Coronal MRI scan of the brain illustrating the frontal and temporal lobes, providing a detailed view of the internal brain structures, particularly useful for evaluating the ventricles and other midline structures.

Radiological investigations were done, and the chest X-ray was normal. CT brain and CT angio brain showed no significant findings. MRI of the brain (Figure 2) showed a hyperintense soft tissue lesion measuring 14 mm × 13 mm × 6.8 mm (craniocaudal × anteroposterior × transverse), which demonstrated moderate homogeneous enhancement with no obvious blooming or diffusion restriction. The lesion involved the right cavernous sinus, adjacent orbital fissures, and the optic canal (Figure 3). It was suspected to be likely of inflammatory etiology. The autoimmune panel test was done, and it came back negative, ruling out any autoinflammatory process. 

In terms of important differential diagnoses, cavernous sinus thrombosis, fungal infection, IgG4-related disease, sarcoidosis, neoplasm, carotid-cavernous fistula, and lymphoma were considered. MR venography and CT venography were performed to exclude cavernous sinus thrombosis, and both showed no evidence of thrombosis. Serum IgG4 levels and serum angiotensin-converting enzyme (ACE) were also measured, and the results were normal, ruling out IgG4-related disease and sarcoidosis. Additionally, chest CT/PET scans were negative, further excluding sarcoidosis. An infectious workup for TB and other potential infections was also conducted, with negative findings on both TB cultures and GeneXpert. All these investigations helped rule out the differential diagnoses, supporting the diagnosis of THS. These findings demonstrate that the thorough diagnostic workup effectively excluded other possible causes of the patient's symptoms.

Treatment

**Figure 4:**
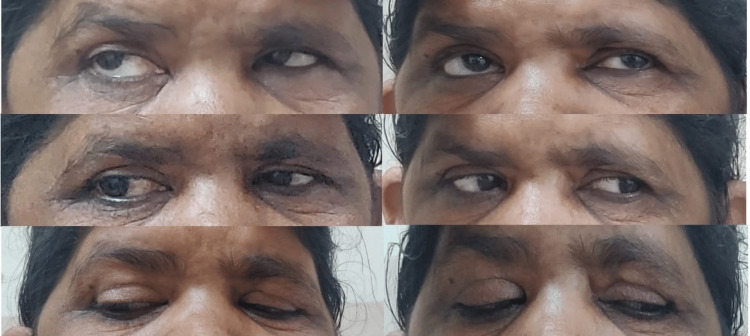
Clinical improvement after methylprednisolone therapy Consent was obtained to publish the image.

Intravenous methylprednisolone (1 mg/kg/day daily) was administered for three days (approximately 60 mg/day as her body weight is 62 kg). This dosage was chosen instead of pulse-dose (1000 mg/day) because the patient did not have any vision-threatening complaints and had moderate disease severity. The clinical timeline of the events has been summarized in Table 1. After three days, a marked reduction in right orbital pain and ptosis was observed, along with improvement in ocular mobility (Figure 4).

**Table 1:** Timeline of clinical events NSAIDs: nonsteroidal anti-inflammatory drugs

Day/time frame	Clinical event	Management/findings	Outcome
Day 1-20	Right-sided headache and eye pain progressed to drooping of the right eyelid, diplopia, and worsening eye pain	Self-managed with paracetamol/NSAIDs, temporary relief, and no formal medical consultation	Symptoms persisted
Day 20	Hospital reporting and admission	Detailed neurological exam revealed III, IV, and VI cranial nerve palsy; sluggish right pupillary reflex	MRI brain showed an enhancing lesion in the right cavernous sinus with orbital apex extension
Day 20–23	Intravenous methylprednisolone (1 g/day for 3 days)	Marked reduction in orbital pain, ptosis, and improved ocular movements	Clinical improvement
Day 23-3 months	Oral prednisolone taper (60 mg → 40 mg → 20 mg → 10 mg at 2-week intervals)	Continued symptomatic improvement	Stable condition and complete resolution of headache

Conclusion and results

Outcome and Follow-Up

The patient was advised to return for follow-up after three days, by which time the symptoms had completely resolved. The glucocorticoid dosage was tapered from 60 mg daily to 40 mg, then to 20 mg, and finally to 10 mg, with reductions occurring every two weeks.

Patient Perspective

From the patient’s perspective, she reported a significant improvement in her condition, with the right eye pain and ptosis alleviating substantially within the first few days of treatment. She mentioned that the reduction in symptoms provided immediate relief, improving her ability to perform daily activities. Throughout the tapering process, the patient felt stable and reported no new or worsening symptoms, expressing satisfaction with the treatment plan and its outcome.

Informed consent

Written consent was obtained from the patient for recording the data and publication of this case report. The patient also consented to the use of images, if required. A copy of the written consent is available for review by the Editor-in-Chief of this journal upon request.

## Discussion

A unique presentation of unilateral headache, painful ophthalmoplegia, and associated with restriction of orbital movements and cranial nerve palsy is likely to be THS. It is an idiopathic neuroinflammatory condition that occurs in approximately one out of every one million people per year [5]. There is no gender bias in the occurrence of THS, and its incidence spans a wide age range, from the first to the eighth decade of life [6]. The main symptoms include orbital pain, which is usually prominent over the frontal and temporal regions, and restriction of movement of the eye due to the palsies of nerves involved, mainly the oculomotor, trochlear, and abducens [5]. One study found that the sixth cranial nerve was the most commonly involved, followed by the third and fourth cranial nerves. However, various other studies have shown varied presentations, with the third nerve being the primary nerve implicated in this condition [7,8].

The imaging shows a lesion in the right cavernous sinus extending toward the optic canal and the superior orbital fissure. There was a sluggish pupillary reflex and partial involvement of the optic nerve on examination, which could be due to this spread. Although THS typically does not involve the optic nerve, involvement of the orbital apex can produce visual impairment and afferent pupillary defects. This case has a unique presentation and falls in the spectrum of cavernous sinus-orbital apex inflammatory syndromes. Highlighting this overlap is important as this underscores the need for the extensive radiological examination and its correlation while diagnosing and treating THS.

THS can be diagnosed as per ICHD-3 diagnostic criteria (Table 2) [9]. A study found an atypical manifestation of THS with visual impairment, in which the inflammation involves the orbital apex, resulting in optic nerve damage. A similar finding was observed in our patient, where the optic nerve was affected and characterized by a sluggish light reflex [10]. It occurs due to inflammation leading to optic disc swelling and, in such cases, the patient may have temporary or permanent loss of visual acuity [5]. Systemic complications have not been reported in THS except in certain conditions where patients present with nausea, vomiting, and fatigue, and this usually occurs due to the intense painful ophthalmoplegia [6].

**Table 2:** The ICHD-3 outlines the diagnostic criteria for THS ICHD-3: International Classification of Headache Disorders, 3rd edition; THS: Tolosa-Hunt syndrome

ICHD-3 criteria for THS
A. Requirement: unilateral orbital or periorbital headache. Presenting case had unilateral orbital or periorbital headache fulfilling criterion C
B. Requirement: Granulomatous inflammation of the cavernous sinus, superior orbital fissure, or orbit demonstrated by MRI or biopsy. Both of the following are in the present case fulfilling the criteria: 1. Granulomatous inflammation of the cavernous sinus, superior orbital fissure, or orbit, demonstrated by MRI or biopsy 2. Paresis of one or more of the Lord, fourth, and/or sixth ipsilateral cranial nerves
C. Headache preceded paresis by ≤2 weeks or developed simultaneously, and was ipsilateral to the lesion. Evidence of causation is demonstrated by both of the following: (1) the headache was ipsilateral to the granulomatous inflammation; and (2) the headache preceded paresis of the third, fourth, and/or sixth cranial nerves by ≤2 weeks or developed concurrently
D. Not better accounted for by another ICHD-3 diagnosis

The etiology of THS is largely unknown. There are several mimics of THS, like pituitary adenoma, orbital apex syndrome, IgG4-related diseases, acute orbital myositis, Gradenigo’s syndrome, trochleodynia, carotid-cavernous fistula, orbital aspergillosis, Burkitt lymphoma metastasis, Miller-Fisher syndrome, Eales disease, Sclerokeratitis, Takayasu arteritis, and myasthenia gravis. Diagnosis of THS is made by eliminating alternative diagnoses and mostly depends on the evidence of inflammation in the MRI brain [8,11]. In this patient, MRI and CT imaging studies were done, ruling out multiple sclerosis, neoplasms, and malignancies. MRI showed an enhancing lesion in the right cavernous and orbital fissure with features suggesting an inflammatory process [11]. 

The patient presented only with painful ophthalmoplegia on the right side and did not exhibit any musculoskeletal or sensory impairment, thereby ruling out Guillain-Barré syndrome and myasthenia gravis. Other autoimmune neurological conditions were excluded based on CSF analysis for antinuclear antibodies (ANA) and other autoantibodies.

Stroke can occur in THS due to inflammation spreading to nearby blood vessels, potentially leading to vascular complications such as thrombosis or hemorrhage [7]. A rare cause of painful ophthalmoplegia was found to be associated with immunoglobulin G4-related diseases [4]. IgG4-related disease is an idiopathic, chronic, multiorgan inflammatory condition [4]. IgG4-related disease is an idiopathic, chronic, multiorgan inflammatory condition. Patients typically present with ocular pain, and its ophthalmologic manifestations are often almost indistinguishable from those of THS [4,12]. It was found that the only differentiation factor of THS from IgG4-related diseases was the absence of IgG4 staining plasma cells, storiform fibrosis, obliterative phlebitis, and systemic involvement [12]. Heart failure can potentially occur in THS patients due to severe inflammation and systemic effects that could lead to stress on the cardiovascular system [9,13].

Glucocorticoids are typically effective in treating THS, resulting in a substantial reduction in pain within a short duration of prescribing glucocorticoids like prednisolone (60 mg daily), and are usually continued for two weeks and then tapered over a month or longer if pain recurs. Sometimes steroid-sparing agents like azathioprine and methotrexate may need to be added [8]. A standard treatment approach utilizes high-dose steroids, with a gradual dose reduction over months or longer, depending on the individual case. It is found that relapses are common and are usually separated by months to years, and hence, patients are counseled regarding follow-up MRI studies [3].

MRI may not provide a conclusive diagnosis of THS due to its lack of specificity. Although characteristic signal alterations, such as hypointensity on T1-weighted images and isointensity on T2-weighted images, are often observed, these findings are not pathognomonic and can mimic other conditions, including meningioma, lymphoma, and sarcoidosis. Considering the inherent limitations of preliminary imaging studies, some experts suggest that the MRI inflammation resolving with systemic corticosteroids could be a determinative criterion for THS [14].

This approach, however, necessitates careful clinical correlation and exclusion of other potential etiologies, as the spontaneous resolution of inflammatory processes can occur in certain conditions [15]. The relatively short follow-up of only three months is a limitation of this case report, and THS is known to relapse after months or years, and longer follow-ups with advanced imaging are required.

## Conclusions

THS is an acute neuroinflammatory disorder that presents with severe ophthalmoplegia, ptosis, and cranial nerve paresis. As a diagnosis of exclusion, meticulous differentiation from other potential etiologies is imperative through comprehensive clinical evaluation, laboratory investigations, and neuroimaging. While the precise pathophysiology remains unclear, THS often exhibits a dramatic and rapid response to systemic corticosteroid therapy, with the potential for spontaneous remission and subsequent recurrence.

This case highlights the importance of early recognition and timely initiation of treatment, which can lead to rapid symptom resolution and improved quality of life. However, as relapses are common, long-term follow-up and repeat imaging remain essential for optimal management. Our report underscores the need for heightened clinical suspicion, comprehensive diagnostic evaluation, and multidisciplinary collaboration in managing patients presenting with painful ophthalmoplegia.

## References

[1] Arnold M (2018). Headache Classification Committee of the International Headache Society (IHS) the International Classification of Headache Disorders, 3rd edition. Cephalalgia.

[2] (2025). Tolosa-Hunt syndrome - an overview. https://www.sciencedirect.com/topics/medicine-and-dentistry/tolosa-hunt-syndrome.

[3] Siddhanta KC, Shreeyanta KC, Kunwar P, Dhungana K (2021). Tolosa-Hunt syndrome: a case report. J Nepal Med Assoc.

[4] Douedi S, Awad M, Shenouda D, Mack P, Carson MP (2020). Tolosa-Hunt syndrome: a non-classical presentation of a rare cause of unilateral headache and painful ophthalmoplegia. J Clin Med Res.

[5] (2025). CARE checklist. https://static1.squarespace.com/static/5db7b349364ff063a6c58ab8/t/5db7bf175f869e5812fd4293/1572323098501/CARE-checklist-English-2013.pd.

[6] Amrutkar CV, Burton EV, Lui F (2025). Tolosa-Hunt Syndrome. http://www.ncbi.nlm.nih.gov/books/NBK459225/.

[7] Ahmed HS, Shivananda DB, Pulkurthi SR, Dias AF, Sahoo PP (2024). Clinical profile and outcomes in Tolosa-Hunt Syndrome; a systematic review. J Clin Neurosci.

[8] Kmeid M, Medrea I (2023). Review of Tolosa-Hunt syndrome, recent updates. Curr Pain Headache Rep.

[9] Kim H, Oh SY (2021). The clinical features and outcomes of Tolosa-Hunt syndrome. BMC Ophthalmol.

[10] Badakere A, Patil-Chhablani P (2019). Orbital apex syndrome: a review. Eye Brain.

[11] Jha S, Tiwari M, Agarwal N, Datta A, Shobhana A (2023). Pituitary hyperplasia in Tolosa Hunt syndrome: demystifying the great mimic. Ann Indian Acad Neurol.

[12] Mathews B, Zehden J, Kostic M (2024). Tolosa Hunt syndrome: an atypical presentation of a rare condition. Duke J Case Rep Ophthalmol.

[13] Msigwa SS, Li Y, Cheng X (2020). Tolosa Hunt syndrome: current diagnostic challenges and treatment. Yangtze Medicine.

[14] Kapila AT, Ray S, Lal V (2022). Tolosa-Hunt syndrome and IgG4 diseases in neuro-ophthalmology. Ann Indian Acad Neurol.

[15] Al-Ezzi SM, Inban P, Chandrasekaran SH (2024). The role of exercise training and dietary sodium restriction in heart failure rehabilitation: a systematic review. Dis Mon.

